# Matrix Metalloproteinases and Their Inhibitors as Potential Prognostic Biomarkers in Head and Neck Cancer after Radiotherapy

**DOI:** 10.3390/ijms25010527

**Published:** 2023-12-30

**Authors:** Gabriel Fornieles, María Isabel Núñez, José Expósito

**Affiliations:** 1 Doctoral Programme in Clinical Medicine and Public Health, University of Granada, 18012 Granada, Spain; gabrielfest@correo.ugr.es; 2 Department of Radiology and Physical Medicine, School of Medicine, University of Granada, 18016 Granada, Spain; jose.exposito.sspa@juntadeandalucia.es; 3 Biopathology and Regenerative Medicine Institute (IBIMER), Centre for Biomedical Research, University of Granada, 18016 Granada, Spain; 4 Biosanitary Institute of Granada (ibs.GRANADA), 18012 Granada, Spain; 5 Radiation Oncology Department, Virgen de las Nieves University Hospital, 18014 Granada, Spain

**Keywords:** head and neck cancer (HNC), matrix metalloproteinases (MMPs), tissue inhibitors of metalloproteinases (TIMPs), prognosis, radiotherapy

## Abstract

Head and neck cancer (HNC) is among the ten most frequent tumours, with 5-year survival rates varying from 30% to 70% depending on the stage and location of the tumour. HNC is traditionally known as head and neck squamous cell carcinoma (HNSCC), since 90% arises from epithelial cells. Metastasis remains a major cause of mortality in patients with HNSCC. HNSCC patients with metastatic disease have an extremely poor prognosis with a survival rate of less than a year. Matrix metalloproteinases (MMPs) have been described as biomarkers that promote cell migration and invasion. Radiotherapy is widely used to treat HNSCC, being a determining factor in the alteration of the tumour’s biology and microenvironment. This review focuses on analysing the current state of the scientific literature on this topic. Although few studies have focused on the role of these proteinases in HNC, some authors have concluded that radiotherapy alters the behaviour of MMPs and tissue inhibitors of metalloproteinases (TIMPs). Therefore, more research is needed to understand the roles played by MMPs and their inhibitors (TIMPs) as prognostic biomarkers in patients with HNC and their involvement in the response to radiotherapy.

## 1. Introduction

Head and neck cancer (HNC) is among the ten most frequent types of tumours, occupying the sixth place. Worldwide, HNC accounts for more than 890,000 new cases and 450,000 deaths annually due to this cancer [1]. It is defined as a malignant neoplasm that is developed from the mucosal epithelium in the oral cavity, pharynx and larynx. There are many types of HNCs, which are categorized by their anatomical location following the International Classification of Diseases (ICD-10) from the World Health Organization (WHO). HNC is traditionally known as head and neck squamous cell carcinoma (HNSCC), since 90% arises from epithelial cells [2,3]. Its incidence has decreased significantly in countries within Asia, North America, Australia, and south and east of Europe. But it is increasing in several countries located in Eastern and Northern Europe among men, and in Southern and Western Europe among women, reflecting the tobacco epidemic. This fact contrasts with the decline in other Western countries where smoking cessation has been earlier. However, an increased incidence in oral cancer in sites related to HPV infection was found, which could be attributed to changes in sexual behaviour, tobacco, alcohol and diet [4,5]. According to EUROCARE, considering head and neck tumours as the most lethal tumours, are those located in the hypopharynx and larynx with 25% and 59%, respectively. This is followed by the oropharynx with 39%, tongue with 43%, oral cavity with 45% and nasopharynx with 49% [6].

Smoking is the most important risk factor for oral cancer. Tobacco consumption has become a global epidemic. One billion men smoke worldwide, with 35% in developed countries and 50% in developing countries. And over 250 million women are smokers, with 22% in developed countries and 9% in developing countries. This trend indicates that men are smoking less and women are increasing their consumption [7]. Alcohol consumption, the second most important risk factor, is associated with the risk of suffering from HNSCC [8]. It is widespread throughout the history of humanity and across the planet. All alcoholic products carry with them an increased risk of HNSCC [9].

Tumour location is one of the most important prognostic factors because it influences cancer’s ability to metastasize. Tumours of the floor of the mouth and those of the posterior 2/3 of the tongue have a greater capacity for migration through lymph nodes than those that originate in the gums and buccal mucosa. Those originating in the lips, both in the vermilion and dermal portions, have a low metastatic behaviour, compared to tongue cancer. However, the location is not the only determining factor and others are important, such as the stage of the tumour [10].

The treatment alternatives are surgery resecting the tumour, radiotherapy (external radiotherapy or brachytherapy or a combination of these treatments) and chemotherapy, depending on the stage at which the oral cancer is found and after evaluating the variables of the tumour size and its possibilities of propagation to distance. Early diagnosis is essential so that therapy is as simple and effective as possible, directly influencing the success of treatment [3].

Locoregional recurrence is a major cause of patient mortality and morbidity. Although radiation eradicates a large proportion of tumour cells, selected groups of tumour cells (clonogens) are able to survive and repopulate the irradiated areas. Tumours are known as radioresistant if recurrences are observed within six months after the first course of radiation. Tumours can become resistant to radiotherapy for several reasons. Cells proliferate very rapidly and recolonise between treatments and/or tumour cells exhibit mechanisms to become resistant to radiation, such as hypoxia (as oxygen is needed to increase the damage of radiotherapy to the cell’s DNA). In these cases, systemic treatment can be added, improving the results of radiotherapy [11]. Radioresistance is also associated with a high degree of molecular heterogeneity. Other possible molecular mechanisms of radioresistance are enhanced DNA damage repair capacity, increased reactive oxygen species (ROS) scavenging capacity, epithelial–mesenchymal transition (EMT) and abnormal regulation of programmed cell death, which have revealed new targets for the therapy of different types of tumours, including oral squamous cell carcinoma (OSCC) [12]. The role of cancer stem cells (CSCs) in radioresistance has also been described [12,13].

Tumour dissemination and metastasis are characteristic processes of the relapse and failure of cancer treatment, representing 90% of deaths attributable to cancer. Metastasis represents the end product of a multistage cellular process, called the metastatic cascade, which implies the dissemination of tumour cells to distant organs with their adaptation to different microenvironments. Each of these events is directed by the acquisition of genetic and/or epigenetic alterations, characteristic of tumour cells or stromal cells, which endow the invading cells with characteristics to generate macroscopic metastases. Recent advances have described what these molecular changes are like, which have an impact on the steps to generate a metastasis, and which can be targeted as a therapeutic target [14].

The aim of this review was to summarise the role of matrix metalloproteinases (MMPs) and their inhibitors (TIMPs) as potential biomarkers of a prognosis and radiotherapy response in patients with HNC. Personalized medicine based on quantifiable biomarkers such as MMPs could generate more reversal strategies to improve cure and survival rates in HNC patients with the possibility of deciding the best therapy for them.

## 2. MMPs and TIMPs

**Figure 1:**
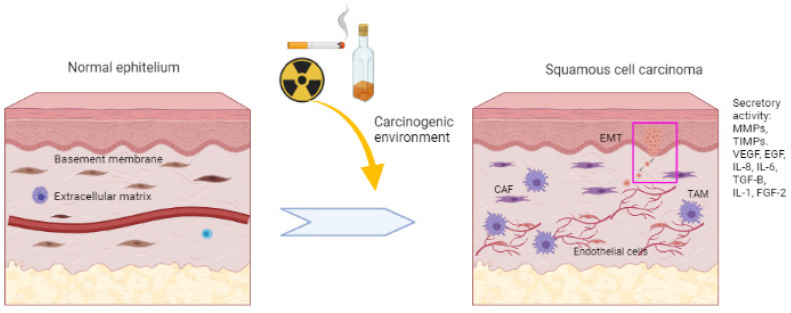
Multistage process of squamous cell tumourigenesis. The mucosa exposed to risk factors with a carcinogenic environment can alter the homeostasis and genetic stability of cells. This leads to increased signalling for cancer progression through uncontrolled cell proliferation and growth. Local invasion and stromal changes are promoted. Growth factors such as FGF-2, EGF and VEGF and inflammatory mediators such as IL-8, IL-2, IL-6, TIMPs and MMPs play a crucial role in BM and ECM breakdown. Decreasing intercellular adhesions and decreasing E-cadherin ends up promoting tumour development and local invasion, which can reach other tissues through blood vessels.

MMPs are a family of zinc-dependent endopeptidases which have the ability to degrade components of the extracellular matrix (ECM). They present a catalytic domain consisting of a zinc ion in the active part, which is essential for metabolic processes, depending on the substrate specificity of the components and their structural characteristics. MMPs are classified into six groups: collagenases, gelatinases, stromelysins, matrilysins, metalloelastases and membrane metalloproteases [15]. MMPs promote tumour invasion and metastasis by degrading the basement membrane (BM), which allows tumour cells to invade surrounding tissue or neighbouring blood vessels (Figure 1). In addition, the degradation of the ECM by MMPs produces the activation of peptides, such as the epidermal growth factor (EGF) which promotes the proliferation of its receptor (EGFR), involved in the signalling of integrin and growth factors, similar to insulin that stimulates tumour progression [16]. Likewise, MMPs cause the overexpression of growth factors such as vascular endothelial growth factor (VEGF), fibroblast growth factor 2 (FGF-2) and transforming growth factor beta (TGF-β), promoting cell proliferation and angiogenesis [17]. In this way, MMPs form an environment that facilitates tumour progression by generating and activating growth factors and other substances [18].

The expression of MMPs is controlled by the tissue inhibitors of metalloproteinases (TIMPs) and in turn, the breakdown of the ECM. The balance between MMPs and TIMPs plays an important role in maintaining the integrity of healthy tissues. The imbalance of these proteins is notable in pathological conditions such as cancer, arthritis and periodontitis [16]. Four different TIMPs have been identified as the following: TIMP-1 (28.5 KDa), TIMP-2 (21 KDa), TIMP-3 (21 KDa) and TIMP-4 (22 KDa). In general, most mesenchymal and epidermal cells are capable of producing TIMPs. TIMP-1, -2 and -3 are produced by white blood cells, and TIMP-4 can be overexpressed in vascular smooth muscle cells. The regulation of MMPs and TIMPs is reciprocal and depends on endogenous growth factors and cytokines. Most MMPs can be inhibited by all four types of TIMPs. The expression of TIMP-1 and TIMP-3 is inducible, while TIMP-2 is constitutive. The presence of TIMP-4 is regulated and restricted depending on the tissue. TIMPs and MMPs are produced by several cell types and it is possible to find them in all bodily fluids like serum, urine, saliva, etc. During pathological or physiological processes, levels can be modified, and these changes could be an approach for diagnosis and prognosis; for example, the MMP-9/TIMP-1 ratio in urine is thought to predict the risk of bladder cancer. Also, there is evidence that could be used for hepatocellular carcinoma and rheumatoid arthritis. Thus, there is some evidence that elucidates the prognostic and diagnostic power of the MMP/TIMP ratio [18].

## 3. MMPs and HNC

### 3.1. Invasion and Metastasis

The role of MMPs in invasion and metastasis has been evaluated by various authors [19,20]. For invasion to occur, a critical step must take place, which is the signalling of the initiation of the metastatic cascade through the interaction of the cells of the tumour with BM. Tumour cells initiate ECM degradation behaviour through BM proteolysis, leading to tumour cell propagation [3,20]. The degradation of the ECM also leads to an important change in the phenotypic change of the cells, acquiring a mesenchymal profile due to a phenomenon called the epithelial–mesenchymal transition, controlled mainly by MMPs [21].

Görögh et al. [20] concluded that MMP-2 overexpression was correlated with metastasis and that high levels of TIMP-1and -2 decrease tumour growth. Ren et al. [22] found that the mRNAs of MMP-7, MMP-13 and MMP-10 were regulated and that MMP-12 and MMP-9 were not regulated in metastatic tumours compared to non-metastatic ones. This suggests that there are genes that play important roles in metastasis through the regulation of MMP-7 and MMP-13. Nishio et al. [23] found that there are higher levels of MMP-2 and MMP-9 at metastatic sites compared to those of the primary tumour. Also, there were statistical differences found in both T1 and T2 cases. As compared to the higher expression of MMPs in metastatic regions, tumour-associated macrophages (TAMs) were in the primary regions. Thus, it can be concluded that the number of TAM and MMP expression levels are expected to have an inverse relationship between the primary and metastatic regions. Chakraborty et al. [24] observed that in 56.2% cases, the expression of MMP-9 was more correlated with the presence of lymph node metastases, an advanced stage of cancer and grade of tumour. De Vicente et al. [25], in an observational study, also concluded that MMP-2 and MMP-9 are overexpressed in patients with lymph node involvement and that MMP-9 is related to a low survival rate and therefore a poor prognosis. Targeting the remodelling of the HNSCC microenvironment has been suggested as a therapeutic approach to preventing lymph node metastasis. In this sense, it has been found that MMP-9 and MMP-14 were upregulated in the metastatic lymph nodes and closely positively correlated with the level of ALG-2 interacting protein X (ALIX), a protein that promotes the migration and invasion of HNSCC cells. The expression of ALIX in HNSCC was analysed and demonstrated to be statistically higher than in normal mucosae. MMP-9 and MMP-14 were found in the cytoplasm of cancer cells and were significantly enhanced in metastatic lymph nodes compared to the primary tumour. The degradation of extracellular matrix caused by MMPs is critical and the results suggest that ALIX could contribute to increased MMP-9 and MMP-14, leading to lymph node metastasis. Data from The Cancer Genome Atlas (TCGA) further verified the correlation between ALIX and MMP-14, although there was no correlation with MMP-9 [26].

### 3.2. Epithelial–Mesenchymal Transition (EMT)

**Figure 2:**
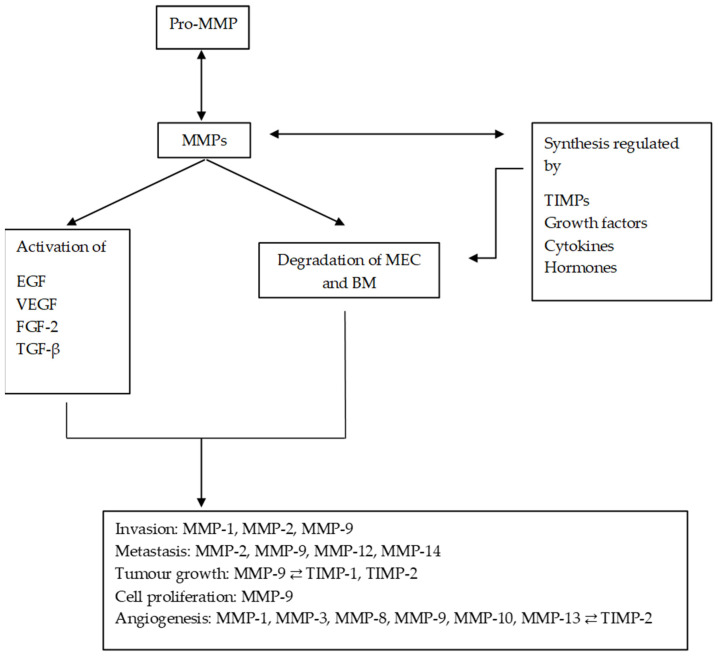
Diagram of the main processes in which MMPs are involved and their regulation by TIMPs. MMPs regulate the synthesis and activation of other substances, promoting and altering the tumour microenvironment. MMPs: matrix metalloproteinases; ECM: extracellular matrix; EGF: epithelial growth factor; VEGF: vascular endothelial growth factor; TGF-β: transforming growth factor beta; FGF-2: fibroblast growth factor 2.

MMPs are a family of endopeptidases responsible for dissolving the ECM and the BM. Figure 2 summarises the main processes in which MMPs are involved. They are produced by various inflammatory and connective tissue cells such as the following: fibroblasts, lymphocytes, endothelial cells and macrophages. And its synthesis is strictly regulated by hormones, growth factors, cytokines and TIMPs [27].

Due to the role played by MMPs in the degradation process of BM and ECM, they have been considered as promising prognostic biomarkers in OSCC. Several studies have shown that the levels of MMPs are increased in cancer patients or patients with oral dysplasia compared to the control group. Smriti et al. [28] obtained a significant increase in salivary MMP-9 in patients with OSCC and oral dysplasia (OD) compared to the control group, and subjects with OD had higher levels than OSCC patients, suggesting greater expression of MMP-9 in direct relation to tumour development. Ghallab et al. [29] established that there was a direct relationship between the levels of MMP-9 and the malignancy of the lesions, for which they designed an observational and cross-sectional study that included 15 controls, 15 OSCC patients and 15 OD patients. MMP-1, MMP-2 and MMP-9 are overexpressed in OD and have the capacity to invade by breaking the BM and the ECM. Also, MMP-1 and MMP-9 were established as biomarkers that promote malignancy by Jordan et al. They studied the expression of MMP-1, MMP-2 and MMP-9 through an experimental study using TaqMan reverse transcription polymerase chain reaction (RT-PCR) in 34 oral dysplasia patients and 15 OSCC patients. The majority of dysplasias became OSCC. The MMP-1 and -9 levels were significantly higher in the OSCC cases compared with the dysplasias (*p* = 0.004 and *p* = 0.01). At the same time, MMP-1 and -9 mRNA levels were also significantly higher in the oral dysplasias that turned into oral cancer compared with those that did not [30].

In addition, Choudhry et al. [31] in a cross-sectional study showed significant increases in MMP-1, MMP-8, MMP-10, MMP-12 and MMP-13 in serum samples of patients with OSCC, compared to the control group, stating that MMP-12 would be the ideal biomarker due to its sensitivity and specificity with a potential diagnosis.

### 3.3. Tumour Growth

An active migration of tumour cells from the original tissue must happen before metastasis and this is carried out by degrading the BM. This membrane constitutes the first barrier for invasion to occur; one of its major components is type IV collagen. MMP-2 is secreted as a zymogen, but in contact with membrane-type matrix metalloproteinase-1 (MT1-MMP) or MMP-14, it is activated [32]. And it begins to degrade BM, which allows for the expansion of the cancer, which subsequently requires an induction of angiogenesis in tumour tissue to nourish proliferating tumour cells [33]. Several studies have associated an overexpression of MMP-9 with the progression of oral cancer and a poor prognosis [33]. Liu et al. concluded that mRNA expression levels of MMPs were significantly higher in HNSCC than in normal tissues, declaring that they could serve as a therapeutic target and prognostic biomarker in HNSCC [34,35].

De Vicente et al. [27] showed how MMP-9 is related to TNM parameters and its expression was associated with a poor prognosis. Zheng et al., through a meta-analysis that involved nine case–control studies that combined 419 patients with oral cancer, demonstrated that there was poorer overall survival in patients who tested positive for MMP-9 compared to those who were negative for MMP-9 in the non-activity-based subgroup. Significant differences in MMP-9 expression were observed between patients with OSCC and metastasis and those without metastasis (*p* < 0.05). Indeed, a significantly higher expression of MMP-9 has been shown in tumours with invasive T3 and T4 stages compared to T1 and T2 tumours [36].

### 3.4. Angiogenesis

Angiogenesis is a critical process in tumour formation and progression. Different signalling cascades have been revealed to be involved in tumour neovascularization, such as VEGF. Hypoxia is a common factor in solid tumours, contributing to cancer progression and poor outcome. A large number of pro-angiogenic factors are regulated by hypoxia, including VEGF, platelet growth factors derived from factor β, the inhibitor of plasminogen activation, etc. [37].

The angiogenic process begins with the recruitment of blood vessels in response to the release of growth factors and cytokines by tumour cells and the secretion of proteases such as MMP-9 [17]. In fact, it is known that the blood vessels of a tumour mass are formed due to various processes, including the appearance of endothelial cells from neighbouring capillaries and small blood vessels in response to the release of growth factors and the recruitment of progenitor cells from the tumour mass of the bone marrow. In addition, tumour cells can also form vascular channels and express endothelial biomarkers. This is a process called vascular mimicry, described by Maniotis et al., and all of these tumour vascularization mechanisms require the activity of MMPs, mainly MMP-1, MMP-2, MMP-9 and MMP-14 [38].

In addition to their pro-angiogenic function, MMPs may also function in anti-angiogenic activity. In fact, a variety of endogenous angiogenic inhibitors are derived from the MMP-mediated degradation of ECM molecules. These inhibitors are angiostatin, a fragment of plasminogen; endostatin, a fragment of type XVIII collagen; and tumstatin, derived from collagen IVα [17]. The accumulation of tumour-associated macrophages (TAMs) in the cancer stroma is correlated with angiogenesis in oral cancer [23]. TAMs also increase the secretion of angiogenic factors such as vascular endothelial growth factor and contribute to the integrity of the vascular structure of the tumour. Nishio et al. found a higher expression of MMP-2 and MMP-9 in the metastatic regions in OSSC patients. These results also showed a greater number of macrophages associated with the primary tumour, which would be responsible for promoting angiogenesis. These results may indicate that the tumour cells in the metastatic region still maintain invasive potential, whereas the TAMs in the primary region may play other roles such as in angiogenesis.

Normal vasculature consists of the interaction of two cell types, endothelial cells and surrounding pericytes, which share a common basement membrane and communicate by physical contact and paracrine signalling. These interactions are necessary for the survival, maturation and stabilization of the vascular system [39]. In contrast, tumour-associated blood vessels show heterogeneous function and anatomy and often become deficient and highly permeable, allowing macromolecules to escape [40]. This occurs in response to the factor responsible for vascular permeability produced by tumour cells. This increased permeability of tumour blood vessels is recognized as a first step in pathological and physiological angiogenesis, which is very similar in healing and inflammation processes [41]. The permeability produced by the structural basis of tumour-associated blood vessels is associated with defects in endothelial cells that form gaps between cells. In addition, defective endothelial cells composed of branches are disorganised and with the loss of intercellular connections, the pericytes that cover the vessels are also abnormal. These abnormalities include the loss of association with the endothelium and an abnormal morphology, associated with deep cytoplasmic projections that enter the tumour [42].

Tumour progression and metastasis depend on the formation and recruitment of new blood vessels in response to the release of proangiogenic factors, such as fibroblast growth factor 2, VEGF and IL-8. However, the overexpression of angiogenic factors such as HGF and placental growth factor (PIGF) has also been shown in the tissue and saliva of patients with OSCC. Moreover, MMP-1, MMP-3, MMP-8, MMP-9, MMP-10, MMP-13 and TIMP-2 were significantly upregulated in the saliva of OSCC patients compared to healthy controls. The concept of a dominance of anti-angiogenic factors, including inhibitors of angiogenesis such as thrombospondin and TIMPs, is replaced by the abundance of pro-angiogenic factors before the initiation of tumour neovascularisation and is known as the angiogenic switch [43].

### 3.5. MMPs as Therapeutic Targets and Prognostic Biomarkers in HNSCC

Several projects have been performed to determine the expression patterns of different MMPs and TIMPs and the associations between their expression and patients’ outcomes in HNSCC. Table 1 summarises the main studies that revealed the correlations between MMP expression and clinical parameters mainly by collecting TCGA data. The results obtained support the roles of some MMPs as diagnostic, prognostic and therapeutic biomarkers in HNC patients [44,45,46,47,48,49].

## 4. MMPs and Radiotherapy

Given that radiotherapy is widely used in the treatment of HNC carcinoma, it would be interesting to analyse state-of-the-art MMPs as biomarkers of prognosis and radioresponse in this pathology.

Many steps of carcinogenesis are regulated by the stromal microenvironment in which tumour cells develop. One of the most relevant changes in the tumour stroma is the activation of fibroblasts, which respond to signals from invading cancer cells by adopting phenotype and functional changes which give them the ability to stimulate cancer cell growth. Activated stromal fibroblasts can secrete various trophic factors and extracellular matrix enzymes which cause alterations and extracellular matrix remodelling [50].

Ionising radiation produces changes in the tumour microenvironment, promoting the malignant behaviour of cancer-associated fibroblasts (CAFs) with the triggering of growth factors such as the in situ activation of TGF-β, which has been involved in the progression of cancer. Therefore, radiation can disturb the stroma, modifying the fibroblast phenotype and being irreversible. Thus, the stroma can serve as a cluster of aggressions due to previously induced stress. All these changes give rise to the promotion of an oncogenic environment that deregulates intercellular interactions and how the extracellular matrix deals with cells of affected tissues [50,51,52].

Fibroblasts acquire an MMP-secreting phenotype after being exposed to low-dose radiation. In breast cancer, Tsai et al. [44] demonstrated an overexpression of MMP-1, -3, -7, -9, -10 and -12 in a three-dimensional culture with fibroblasts and epithelial tumour cells after hypofractionated radiotherapy. These authors suggested that stromal fibroblasts acquire secretory activity after radiotherapy, providing evidence for the matrix degradation by MMPs, representing an important mechanism by which ionizing radiation-induced fibroblast senescence promotes growth of surrounding epithelial cells [50].

Mueller and Schultze [51], in a study on complications in reconstructive surgery in OSCC patients who were previously irradiated, found that in non-irradiated grafts, TIMP-1 was expressed by 38.7%. In grafts with adjuvant radiotherapy, an increase in TIMP-1 was observed, expressing itself in 80.3%, and in patients with radiochemotherapy, 82.3% expression of TIMP-1 was described. A significant difference was found between the groups that did and did not receive radiotherapy (*p* = 0.003), while no significant difference was found with the group with adjuvant radiotherapy (*p* = 0.727). MMP-1 was also studied and no difference was found between any of the three groups. One common late effect of radiation is fibrosis. Radiation-induced fibrosis is the excess accumulation of extracellular matrix component proteins yielding reduced tissue compliance. The induction of TIMP-1 has been associated with the suppression of ECM-degrading pathways promoting fibrosis in the irradiated graft bed prior to surgery. This fibrotic situation generates healing problems and complications in clinical detectable flaps [50].

Other authors have studied the mobility and invasiveness of tumour cells after radiotherapy. Artacho-Cordón et al. [46] obtained preliminary results in MCF-7 cell lines, through which there was proteolytic activity after 6 Gy. MDA-MB-231 cells activated their gelatinolytic function, favouring radioresistance and invasiveness of breast cancer cells, which could explain their aggressive phenotype. It has been documented that basic molecular changes occur in human vocal fold tissue following irradiation and also that migration and invasive capacity increase after sublethal doses of radiation [53,54,55]. Increased MMP-2 activity potentiates migration and invasiveness after sublethal irradiation. Moreover, it has been suggested that the use of higher single fractions in radiotherapy favours radio-induced clonogenic cell death instead of migration and invasion in some tumours [50]. Other results have shown that irradiation promotes the recruitment of stem and progenitor cells from the bone marrow niche mediated by MMP-9 [55].

An increase in the levels of MMP-2 and MMP-9 has been described in the irradiation of various tumours. Some authors have found an increase in the expression of the MMP-1 gene after one hour of fractionated radiotherapy in laryngeal tumours, producing vocal fold fibrosis [53]. MMP-9 also increased in meningiomas after radiation. Heissig et al. described how MMP-9 was deregulated after radiation and observed how VEGF increased dependent upon the increase in MMP-9 after low-dose radiotherapy in breast cancer cells [55]. Likewise, there are several authors who have focused their research on the expression of MMPs and TIMPs during RT. Many researchers have reached the same conclusion from different starting points. The study performed by Virós et al. showed that patients with a high expression of MMP-9 had a six-fold risk of dying as a result of disease progression compared to patients with low MMP-9 expression. After radiotherapy or chemoradiotherapy, the 5-year local relapse-free survival was 84.8% for the group of patients with a low MMP-9 expression level and 55.2% for the patients with high MMP-9 expression levels (*p* = 0.006). Nevertheless, there were no significant differences in MMP-2 and MMP-9 expression in cancer tissue with respect to the primary location, cancer extension, histological grade or type of treatment. These authors concluded that MMP-9 is altered during treatment with radiotherapy and chemotherapy, with overexpression being a poor prognostic factor in terms of survival and local control [56].

The p53 gene was found to express multiple p53 splice variants (isoforms) in a physiological, tissue-dependent manner. P53 plays an important role in the response of ionising radiation [57]. Some investigations have compared the radiosensitivity of cell lines containing both TP53 mutations and the deletion of the fragile histidine triad (FHIT, the gene most commonly associated with 3p deletion) to “single-hit” lines with only TP53 mutation. MMP-2 and MMP-9 activity was also analysed between xenograft tumours derived from “double-hit” and “single-hit” cell lines. The researchers found that xenograft tumours from “double-hit” cell lines had increased MMP-2 and MMP-9 activity compared to tumours from “single-hit” cell lines, which could also account, in part, for the poor clinical outcome of HNSCC patients with the “double-hit” profile [58].

In a more recent investigation, a statistically significant relationship in TIMP-1 levels was found between breast cancer patients who received radiotherapy to their lymph nodes and those whose lymph nodes were not irradiated. Significant correlations were also found between MMP-3 and TIMP-4 levels, and some variables related to patient characteristics and tumour biology, such as menopausal status tumour classification, differentiation grade and E-cadherin presence. Furthermore, a statistically significant correlation was documented between MMP-9 and TIMP-3 levels with the type of radiation toxicity. This research showed that radiotherapy significantly reduced TIMP-1 and -3 levels. The authors also suggested that MMP-9 and TIMP-3 could be proposed as predictors of radiotherapy toxicity in breast cancer, accepting that they could be useful prognostic biomarkers for this pathology [59].

Significant increases in active mouthrinse matrix metalloproteinase-8 (aMMP-8), a potential indicator of periodontal destruction, were also found in HNC patients after radiotherapy [60]. Other authors have studied the usefulness and ability of the mouthrinse aMMP-8 immunoassay and IL-6 levels (ELISA) to diagnose and characterise possible radiotherapy-induced deterioration of oral and periodontal tissues in HNC. These results showed that RT was associated with both impaired periodontal health and elevated levels of aMMP-8 in HNC patients. The most prominent changes in clinical periodontal measures were observed in clinical attachment loss (CAL), which corresponded to a rapid progression (from grade A to grade C) of periodontitis according to the periodontitis classification system [61]. At the same time, radiotherapy had a significant impact on the mean levels of aMMP-8 in the mouthrinse. Levels of aMMP-8 increased significantly during the six weeks of radiotherapy and decreased after radiotherapy, although not significantly when measured one month after the end of radiotherapy [62]. The elevated levels of aMMP-8 observed in HNC patients after radiotherapy suggest an increased susceptibility to further periodontal degeneration and the need for specific periodontal prevention and treatment. These results suggest the potential benefits of point-of-care MMP-8 testing for screening and monitoring periodontal health status over time in patients undergoing cancer treatment [57,58,59,60,61,62].

All of the above described demonstrates the influence of radiation on the alteration of the tumour microenvironment. In addition, radiation plays a role in the activation of MMPs, which could be involved in tumour growth, angiogenesis and dissemination, suggesting an increased risk of metastasis in cells surviving radiotherapy.

## 5. Conclusions

The MMP family is involved in tumour proliferation, EMT, angiogenesis, invasion and metastasis in HNC. Results published to date suggest that patients with HNC may express distinct profiles of MMPs depending on the metastatic stage of the cancer, primary tumour site, type of tissue form which the tumour originated or genomic differences between patients.

Radiotherapy is a common modality of treatment for HNC. It has been observed that various MMPs are upregulated by radiation and their expression has been associated with a poor prognosis of patients with HNC. Although the mechanistic value of the MMP family as a therapeutic target and prognostic biomarker in HNC has not been fully elucidated, the main results obtained so far support the role of some MMPs as diagnostic, prognostic and therapeutic biomarkers in HNC patients.

## Figures and Tables

**Table 1:** Prognostic utility of MMPs and TIMPs in patients with HNSCC.

Author	Biomarker	Database	N	Tumour	Conclusions
Hauff et al., 2014 [44]	MMP-2/-9	TCGA	377	HNSCC	Human HNSCC tumours show increased mRNAs of MMP-2 and MMP-9. In HPV+ tumours, MMP-2 and MMP-14 expression correlates with a worse 5-year survival.
Sáenz-de-Santa-María et al., 2020 [45]	MMP-2/FAK	TCGA	543	HNSCC	The invasion is tuned by a balance between FAK and MMP-2. Invasive cancer overexpresses the MMP-2 gene.
Liang et al., 2020 [46]	MMP-25	TCGA	502	HNSCC	Expression levels of MMP-10, MMP-19, MMP-24, MMP-25 are associated with prognosis. MMP-25 is associated with prognosis and immune infiltration.
Wu et al., 2021 [47]	PLAU1 and MMP-1	TCGA	80	HNSCC	PLAU1 functions as an oncogenic driver to regulate MMP-1, affecting invasion and proliferation.
Hingorani et al., 2021 [48]	MMP-2, -9	TCGA	350	HNSCC	MMP-2 and MMP-9 gene expression is correlated with perineural invasion.
Cai et al., 2022 [43]		TCGA/ Cancer SEA	519	OSCC/ HNSCC	MMP-1, -3, -8, -9, -10, -13 and TIMP-2 are overexpressed and suggested as prognostic biomarkers of OSCC.
Zhang et al., 2022 [35]	MMP-1	TCGA	103	HNSCC	MMP-1 is correlated with advanced tumour size, node metastasis and an advanced pathological grade and lower patients’ survival.
Liu et al., 2023 [49]	MMP-1, -2, -3, -7, -9, -10, -11, -12, -13, -14, -17, -20 and -28	TCGA/Oncomine	439	HNSCC	MMP-14, -16 and -19 are significantly correlated with the clinical prognosis of HNSC patients.

TCGA: The Cancer Genome Atlas; HNSCC: head and neck squamous cell carcinoma; FAK: focal adhesion kinase; PLAU1: urokinase-type plasminogen activator.

## Data Availability

Not applicable.
